# One‐Year Clinical Evaluation of the Horizontal Mattress Sling Suture for Aesthetic Crown Lengthening

**DOI:** 10.1155/crid/9986106

**Published:** 2025-12-22

**Authors:** Najla S. Kasabreh, Hearos A. Bedros, Dimitris N. Tatakis

**Affiliations:** ^1^ Department of Oral and Maxillofacial Surgery, Oral Medicine and Periodontology, College of Dentistry, University of Jordan, Amman, Jordan, ju.edu.jo; ^2^ Department of Periodontics, School of Dental Medicine, Case Western Reserve University, Cleveland, Ohio, USA, case.edu; ^3^ Department of Periodontology, College of Dentistry, The Ohio State University, Columbus, Ohio, USA, osu.edu

**Keywords:** case reports, crown lengthening, dental, esthetics, sutures, wound healing

## Abstract

**Introduction:**

In aesthetic crown lengthening (ACL) surgery, conventional suturing techniques, such as interrupted or vertical mattress sutures, involve piercing both buccal and palatal tissues. This preliminary case series introduces a new suturing approach, “the horizontal mattress sling suture” (HMSS), that eliminates the need to pierce palatal tissues and thus avoids the requirement for palatal anesthesia.

**Clinical Consideration:**

Three healthy patients with excessive gingival display sought treatment at the Department of Periodontics at the University of Jordan. ACL surgery was performed following comprehensive dental and radiographic examinations, as well as periodontal prophylaxis. The HMSS, which combines the horizontal mattress and sling suture techniques, was employed to secure the buccal flap. Follow‐up visits were scheduled at 2 weeks and at 3, 6, and 12 months postsurgery.

**Results:**

All three cases showed uncomplicated healing with no adverse effects. Patients experienced manageable pain during the initial days postsurgery, readily controlled with routine analgesics. None of the patients reported any complications or expressed any concerns, and all were satisfied with the outcomes at each follow‐up visit.

**Conclusion:**

HMSS, a suturing technique designed for single‐flap procedures, such as ACL, is a promising approach that provides a better patient experience by avoiding the necessity to anesthetize the corresponding aspect of the oral soft tissues where a flap was not elevated.

## 1. Introduction

Crown lengthening (CL) is a surgical procedure designed to increase supragingival tooth structure, primarily for restorative purposes [1, 2]. It can also be performed for aesthetic reasons [2, 3]. Functional CL is indicated when planned restorative treatment requires the establishment of the ferrule effect or an increase in crown height. Aesthetic crown lengthening (ACL) is performed to address uneven gingival contours, short clinical crowns, or altered passive eruption, which may result in excessive gingival display (EGD), commonly referred to as “gummy smile”; the treatment of EGD with ACL results in improved smile aesthetics [4] and high patient satisfaction [5].

There are different treatment options that can be used to increase the crown length, such as orthodontic supraeruption, gingivectomy, with or without osseous surgery. Such a procedure is aimed at re‐establishing and/or avoiding invasion of the supracrestal soft tissue attachment, regardless of whether further reconstructive/restorative treatment is needed. When ostectomy and/or osteoplasty are indicated, flap elevation is necessary. Both buccal and palatal/lingual flaps need to be elevated for functional CL. In contrast, for ACL, typically only the buccal flap needs to be reflected to allow the performance of required bone reduction; thus, anesthesia of the buccal aspect is necessary for flap creation and elevation, bone reduction, and suturing of the flap to the desired position. Despite no incision or flap elevation being performed on the palatal aspect, the palatal tissues are usually also anesthetized for suturing purposes. The most commonly used suturing techniques during ACL are single‐interrupted sutures [6] and vertical mattress sutures [7].

Single‐interrupted sutures are typically chosen due to their simplicity, broad applicability, effectiveness, and modular nature, which prevents loss of wound closure or flap position control when only one suture is lost. However, closing a flap extending over many teeth with single interrupted sutures is relatively time‐consuming [8].

Vertical mattress sutures have been used for ACL to adapt the flap tightly to the desired position [9]. Vertical and horizontal mattress sutures have been used in ridge augmentation and root coverage procedures to stabilize coronally positioned flaps and reduce tension [10, 11], partly because they allow precise flap edge placement and control [12]. They also allow for suture material placement away from the incision line, thus minimizing interference with the wound [13]. Both aforementioned suturing techniques require penetrating the labial/buccal gingiva and the palatal/lingual gingiva.

In contrast to single interrupted or horizontal/vertical mattress sutures, sling sutures do not pierce both aspects of the soft tissues. Consequently, sling sutures require anesthetizing only the elevated flap side and avoid the need to anesthetize the other aspects of the tissues. Furthermore, sling sutures are a dependable technique that can control flap position because tissues are anchored to the teeth, a nonyielding, immobile structure [12].

Anesthetic injections in the palate are more painful than in other intraoral sites because palatal tissues are bound tightly to the underlying bone and offer little room for expansion upon the introduction of a liquid [14]. This may cause a traumatic experience for many patients, which can cause dental phobia, making patient management difficult, if not impossible, in the dental office. A fear of dental injections is associated with avoidance of dental care in nearly one in 20 people [15, 16]. Due to patients′ negative experiences with palatal anesthesia, many dentists attempt to avoid it whenever possible. In this context, the option of a suturing approach that can eliminate the need for palatal anesthesia benefits both patients and practitioners.

This case report describes a novel suturing technique for stabilizing ACL flaps from the buccal aspect only, without needing to pierce (and anesthetize) the palatal gingiva. The technique applies a combined suturing approach, utilizing a horizontal mattress sling suture (HMSS), whose objective is to appropriately adapt and secure the position of the ACL full‐thickness buccal flap. This report will detail the suturing technique and the application of HMSS in three patients who underwent ACL.

## 2. Clinical Presentation, Case Management, and Clinical Outcomes

### 2.1. Patients′ Demographic, Medical, Dental, and Social History

All patients were medically fit, without any known drug allergies, and abstained from alcohol consumption. The patient in Case #3, who smoked e‐cigarettes socially, ceased vaping a week before and for 2 weeks after the procedure. Additionally, all patients underwent a comprehensive dental examination during the initial visit, revealing no dental or oral soft tissue pathologies.

### 2.2. Case #1

A 25‐year‐old female presented to the periodontal department at the University of Jordan in February 2020, following the completion of orthodontic therapy, with a chief complaint of unhappiness with her gummy smile (Figure 1a). Following examination, the patient was diagnosed with altered passive eruption (Figures 1a and 2a). The patient accepted the proposed ACL treatment and signed a written informed consent form.

Figure 1Clinical extraoral images of Case #1. (a) Preoperative smile. (b) Postoperative smile at 12‐month follow‐up.

**Figure:**
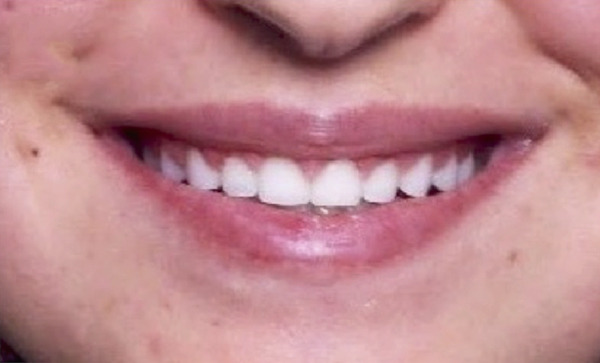
(a)

**Figure:**
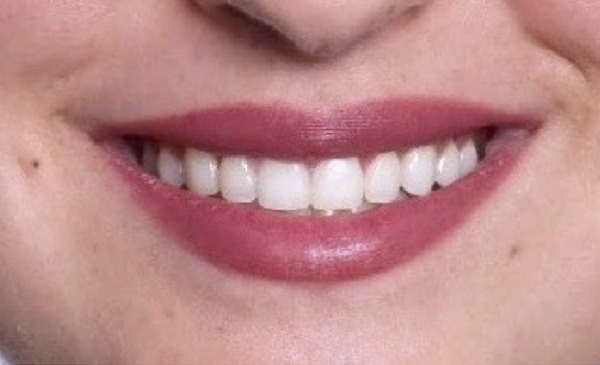
(b)

Figure 2Clinical intraoral images of Case #1. (a) Preoperative retracted view. (b) Aesthetic crown lengthening was completed from the upper right second premolar to the upper left second premolar. Horizontal mattress sling sutures were used to secure the flap. (c) Three‐month postoperative view. (d) Postoperative view at 12‐month follow‐up.

**Figure:**
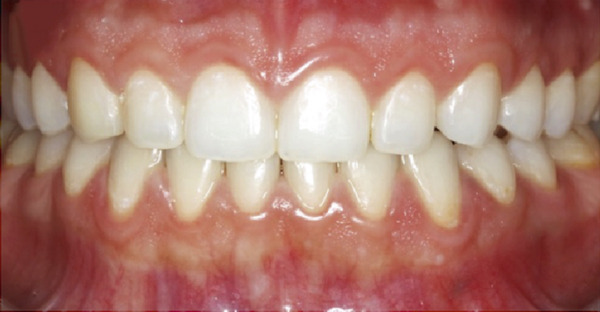
(a)

**Figure:**
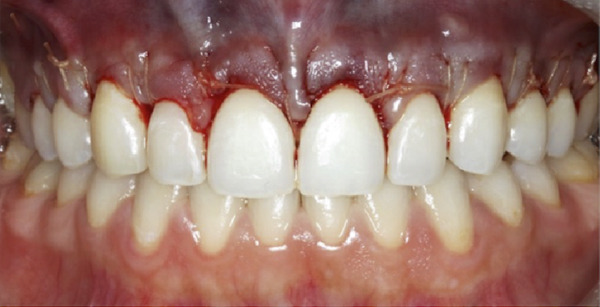
(b)

**Figure:**
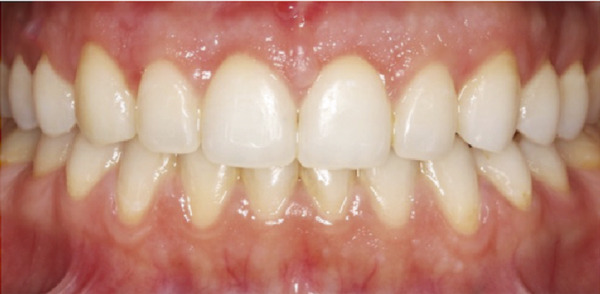
(c)

**Figure:**
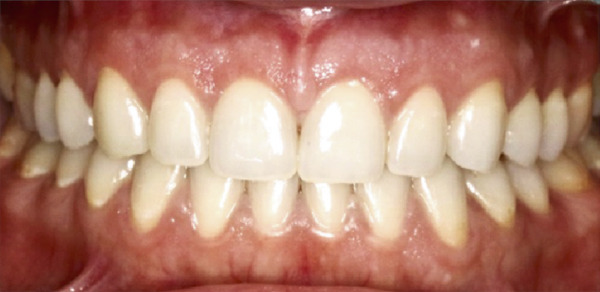
(d)

The treatment started with periodontal prophylaxis. Two weeks later, an appointment for ACL was scheduled. Anesthesia of the buccal tissues, from upper right second premolar to upper left second premolar, was achieved using 4% articaine with epinephrine 1:100,000. Submarginal incisions were performed to remove 1–2.5 mm of soft tissue from each tooth (customized according to cementoenamel junction position). Subsequently, a full‐thickness envelope flap was elevated to allow access to the alveolar crest and perform the needed osseous reduction (osteotomy and osteoplasty). After the completion of osseous corrections, 5‐0 PGA suture material with a noncutting needle was used to adapt the flap using the HMSS technique (Figure 2b).

**Figure 3:**
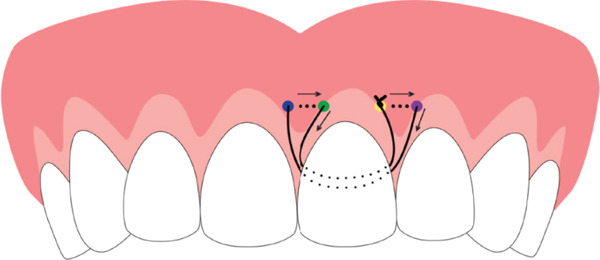
Graphic illustration of the horizontal mattress sling technique. The needle is initially inserted on the tooth′s distal aspect (yellow dot), moved horizontally, and exits the flap toward the distal point (purple dot). The suture is pulled palatally, passed under the contact point between the central and lateral incisors, slung around the central incisor, and drawn toward the buccal aspect under the contact point between the two central incisors. The needle then penetrates the flap from the corresponding more distant mesial point (blue dot). After moving horizontally, it exits the flap from the point near the mesial of the tooth (green dot). The suture is then slung back to the first entry point, and the knot is tied. The order of entry and exit points may be reversed.

The HMSS technique is explained in detail in Figure 3. The needle is inserted from the outer surface of the buccal flap, close to the distal line angle of the maxillary left central incisor. The needle then moves horizontally and exits the flap distal to the entry point, close to the mesial line angle of the maxillary left lateral incisor, keeping it in the same interdental papilla area. The suture is then passed under the contact point between the central and lateral incisors, slung around the central incisor, and drawn toward the buccal aspect under the contact point between the two central incisors. The needle then penetrates the flap close to the mesial line angle of the maxillary left central incisor and exits, after traveling horizontally, close to the mesial line angle of the maxillary right central incisor. The suture is then slung back to the first entry point, and the knot is tied. Accordingly, the two papillae, one between the right central and lateral incisors and one between the two central incisors, are secured simultaneously. The exact process can then be repeated around other involved teeth.

Postoperative instructions were given to the patient, including avoiding gargling/rinsing, engaging in heavy exercise for 24 h, or brushing the surgical site for 2 weeks. Additionally, the patient was advised to use cold compresses (such as an ice pack) and to consume only soft, cold/room temperature foods and beverages. Paracetamol (500 mg, three times a day for 3 days) and chlorhexidine mouth rinse (twice daily for 14 days) were prescribed. The tissues healed uneventfully, and the sutures were removed at 2 weeks, at which time the patient reported no complaints. Additional follow‐up appointments were scheduled at 3 months (Figure 2c) and 12 months (Figure 2d). The remainder of the postoperative course was unremarkable. The patient expressed satisfaction with the surgical outcome and the new appearance of her smile (Figure 1b).

### 2.3. Case #2

A 22‐year‐old female presented to the clinic in November 2022, unsatisfied with her smile (Figure 4a). Following clinical (Figure 5a) and radiographic assessment, treatment plan presentation, and written informed consent, ACL was performed from the upper right second premolar to the upper left second premolar, as detailed in the previous case, to treat the diagnosed uneven gingival margins and altered passive eruption. Flap stabilization was achieved using HMSS. The postoperative protocol was as described in Case #1, and the patient′s postoperative course was uneventful, with no reported complaints. Sutures were removed at 2 weeks (Figure 5b). Follow‐up appointments at 6 months (Figure 5c) and 12 months (Figure 5d) confirmed successful surgical outcomes. The patient expressed satisfaction with the procedure outcomes and the aesthetic improvement in her smile (Figure 4b).

Figure 4Clinical extraoral images of Case #2. (a) Preoperative smile. (b) Postoperative smile at 12‐month follow‐up.

**Figure:**
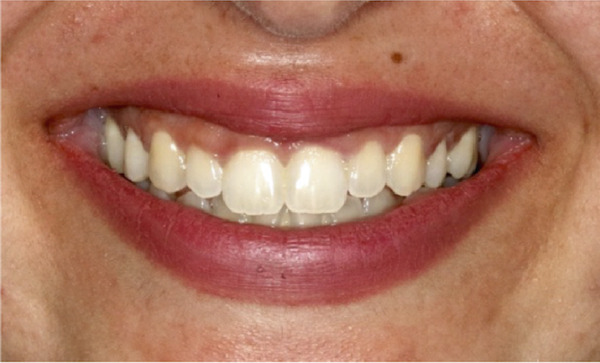
(a)

**Figure:**
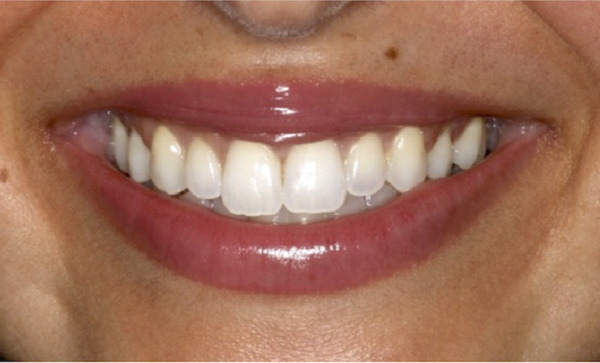
(b)

Figure 5Clinical intraoral images of Case #2. (a) Preoperative retracted view. (b) Two‐week follow‐up after aesthetic crown lengthening was completed from the upper right second premolar to the upper left second premolar. Horizontal mattress sling sutures were removed prior to image taking. (c) Six‐month postoperative view. (d) Postoperative view at 12‐month follow‐up.

**Figure:**
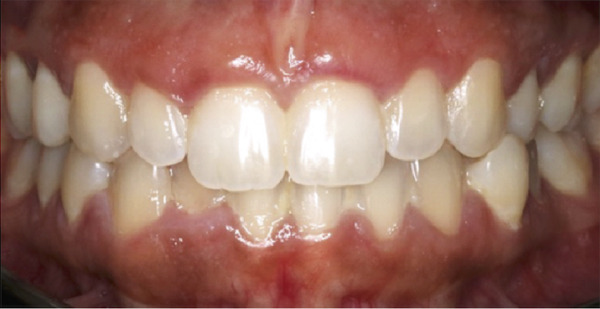
(a)

**Figure:**
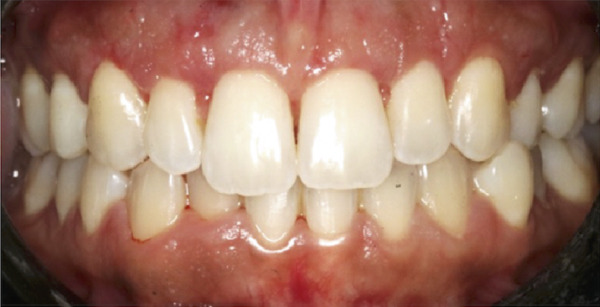
(b)

**Figure:**
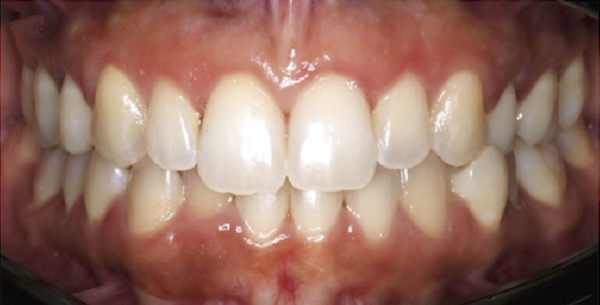
(c)

**Figure:**
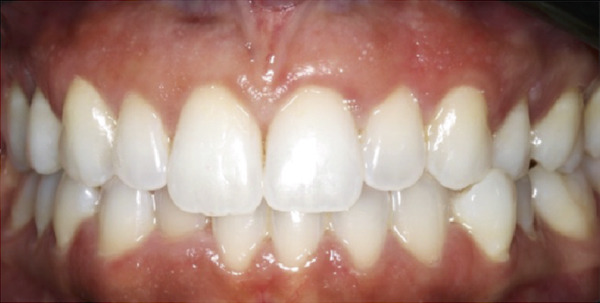
(d)

### 2.4. Case #3

The restorative department referred a 30‐year‐old female to the periodontal clinic in September 2022 with concerns regarding uneven gingival margins, gummy smile, tooth color, and spacing (Figure 6a). After clinical (Figure 7a) and radiographic assessment, the following findings were discussed with the patient: gingival health, uneven gingival margins, EGD, and malocclusion. The treatment plan accepted by the patient included ACL and veneers; the patient refused orthodontic therapy.

Figure 6Clinical extraoral images of Case #3. (a) Preoperative smile. (b) Postoperative smile at 12‐month follow‐up.

**Figure:**
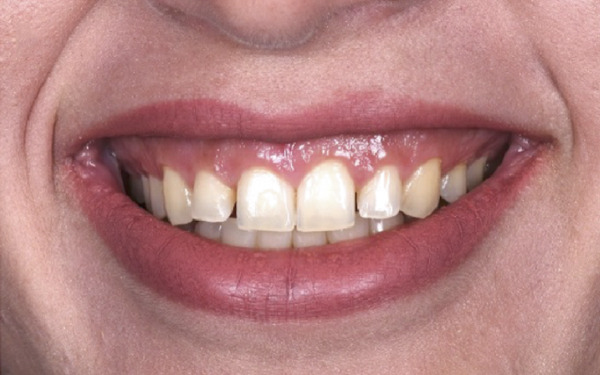
(a)

**Figure:**
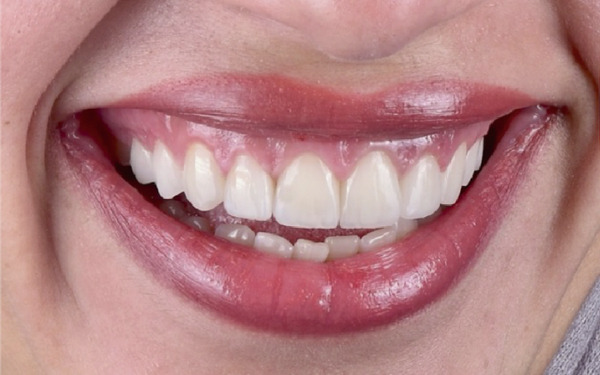
(b)

Figure 7Clinical intraoral images of Case #3. (a) Preoperative retracted view. (b) Aesthetic crown lengthening was completed from the upper right second premolar to the upper left second premolar, using horizontal mattress sling sutures to secure the two independent buccal flaps (full‐thickness flap elevated from the upper right second premolar to the upper right canine; a separate flap was correspondingly elevated on the left side). Only gingivectomy was performed on the incisors. (c) Six‐month follow‐up, during which veneer preparation was performed; retraction cord placement is evident around the teeth in preparation for impressions. (d) Postoperative view at 12‐month follow‐up.

**Figure:**
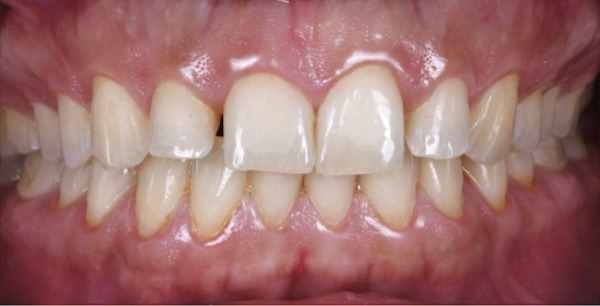
(a)

**Figure:**
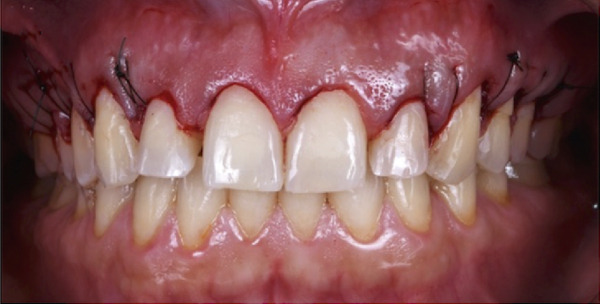
(b)

**Figure:**
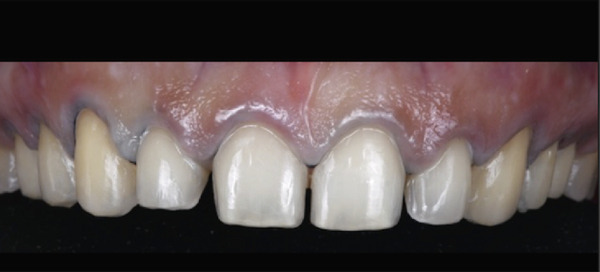
(c)

**Figure:**
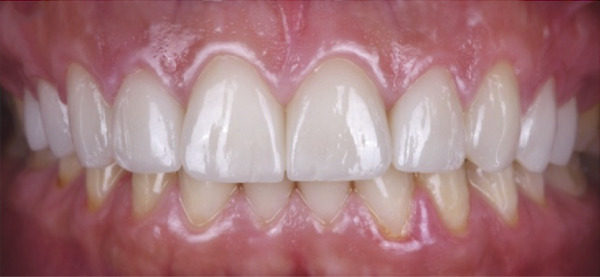
(d)

ACL was performed from the upper right second premolar to the upper left second premolar. More specifically, only gingivectomy was performed on the incisors; additionally, two independent buccal flaps were elevated from the mesial of the right first molar to the mesial of the right canine and correspondingly on the left side to perform the necessary ostectomy and osteoplasty. Flap closure was completed with HMSS (Figure 7b), and the patient was placed on the previously described postoperative protocol. She had no complaints at 2 weeks postoperatively (suture removal). Veneers were placed 6 months after the ACL procedure (Figure 7c was taken at the time of impression making, which was 6 months postoperatively). The patient was followed up for 12 months (Figure 7d). She healed uneventfully and was very satisfied with the aesthetic improvements to her smile (Figure 6b).

## 3. Discussion

The present report is intended to introduce and detail a new suturing approach for single (buccal or lingual) flap procedures. When applied during ACL procedures, the HMSS eliminates the need to engage and therefore anesthetize the soft tissues on the opposite aspect of the alveolar process, providing excellent control of flap position. Consequently, when only a buccal flap is elevated, the HMSS approach offers this advantage over other suturing options. Avoiding palatal tissue penetration eliminates the need for palatal anesthesia and minimizes patient discomfort during suturing and suture removal. All the patients reported no significant outcomes and were satisfied. To the authors′ knowledge, this is the first report to describe this suturing approach.

For any surgical procedure, incision design, flap manipulation, and suturing techniques are essential for proper healing and ideal outcomes. Appropriate suturing protocols are critical for optimal periodontal plastic surgery outcomes [17]. Flap healing starts with clot formation, eventually leading to epithelial and connective tissue attachment to the root [18, 19]. During early healing, flap attachment strength is zero and gradually increases during the first 2 weeks [18, 20], making sutures necessary during this early postoperative period. Suturing principles should be respected in every surgical procedure, including avoidance of excessive tension, proper knot placement, and using the least number of sutures [21]. Even when such principles are respected, some suturing techniques may result in sutures slipping easily between the tooth and flap, thereby impinging on the epithelial and connective tissue attachment. The introduction of suture material in the gingival sulcus may lead to plaque accumulation and an ensuing inflammatory response [22], which could be detrimental to postoperative wound healing and surgical outcomes [23]. In the current suturing technique applied to ACL procedures, the palatal tissues remain intact, which minimizes the potential effect of a possible approximation or introduction of the sling portion of the suture to the palatal sulci.

The concept of applying a combination of suturing techniques is often used in periodontal plastic surgery procedures. For example, vertical mattress sutures have been combined with an anchoring suture technique around splinted contact points [24] and with a sling suture technique around teeth [25], both for coronal tissue displacement. The HMSS presented here is a novel combination approach that utilizes horizontal mattress suturing with slinging around the teeth to secure tissues that are neither coronally advanced nor apically positioned, thereby explicitly avoiding the need to anesthetize the palatal tissues.

Among surveyed periodontists, the most used suturing techniques for CL and other resective procedures are, in decreasing order, the interrupted simple‐loop suture (used by 41.5% of periodontists), interrupted vertical mattress (21%), continuous sling (17%), and continuous vertical mattress sling (9.2%) [21]. The single interrupted suture can be used in all periodontal surgical procedures, such as plastic, regenerative, osseous, and implant‐related surgeries. Its simplicity and ability to well secure the intraoral flaps make it the preferred suturing technique. Whether vertical or horizontal, mattress sutures are primarily used to approximate two flap edges to attain tension‐free primary wound closure. The HMSS offers an additional option beyond these routinely used techniques. Compared to those techniques, HMSS results in fewer knots applied, better tissue stability (due to double sling anchorage around teeth), and likely better tension distribution to the tissues. Thus, HMSS satisfies the aforementioned suturing principles [21]. In addition, since a single HMSS secures two adjacent papillae, less time might be needed to complete the suturing of an extended flap compared to the performance of simple interrupted sutures. The HMSS may find applications beyond the ACL, such as other procedures where only a buccal/lingual flap is elevated and repositioned/coronally positioned.

A recent systematic review concluded that CL surgery results in stable periodontal tissues over time, according to acceptable periodontal healing parameters [26]. Several variables affect ACL outcomes. The surgical approach is crucial, encompassing the amount of soft tissue removed, flap elevation, bone reduction, flap margin position, and suturing technique. The flap should be in a position that accounts for soft tissue healing and supracrestal soft tissue attachment reformation [27, 28] and is sufficiently secured to allow minimal to no position changes throughout the healing period. Complete immobilization of soft tissue wounds in the oral cavity is rarely possible during the postoperative period. Thus, precise and stable suture closure is crucial for successful wound healing [8]. The three patients reported here were followed for up to 12 months, and surgical outcomes were successful, with no significant postoperative changes noted. This suggests that the chosen HMSS technique provides adequate flap stability and proper flap adaptation following ACL.

A potential limitation of HMSS is that it does not offer direct fixation of the elevated papilla tips. However, applying HMSS has not resulted in any complications or aesthetic compromises in the cases where it has been used (Figures 2, 5, and 7). Another limitation would be the presence of open contacts between the teeth. Such open contacts could prevent the suture from benefiting from the anchorage around the teeth. This, however, might be easily resolved by applying a composite material between the teeth before suturing the flap. At the suture removal appointment, the composite material could also be removed. Another limitation of the application of HMSS could be that it is not suitable for cases where there is a lack of adequate keratinized tissue width, which requires apical flap positioning (instead of gingivectomy); this, in turn, could make use of the HMSS both difficult and possibly ineffective for securing the flap at the desired position. A periosteal vertical mattress suture would be a better option in such cases. Sound clinical judgment should always guide the surgeon′s technique choices.

The available evidence in support of this novel suturing technique is limited, suggesting that adequately designed clinical research is needed. This could include a randomized clinical trial to validate the potential superiority of this novel technique over the routine suturing approaches from a wound healing perspective, a patient‐reported outcomes perspective, and an operator‐reported outcomes perspective.

## 4. Conclusion

HMSS is a combined suturing technique that avoids penetration of and the need to anesthetize palatal tissues, provides excellent flap anchorage, and can be successfully applied to ACL cases with adequate keratinized tissue. Within the limitations of a case series, this report suggests that the HMSS technique is a promising suturing alternative for ACL. Future studies could help determine the full extent of this novel suturing technique′s applicability, as well as its advantages and disadvantages.

## Ethics Statement

Ethical approval was not required for this case series of three patients, as it does not meet the definition of human subjects research requiring institutional review board oversight. Written informed consent was obtained from all participating patients prior to inclusion.

## Consent

Written informed consent for the publication of anonymized clinical data and images was obtained from all patients.

## Disclosure

The views expressed in this manuscript are those of the authors.

## Conflicts of Interest

The authors declare no conflicts of interest.

## Author Contributions

Najla S. Kasabreh contributed to the study concept and design, clinical care of the cases, data collection and interpretation, and manuscript drafting. Hearos A. Bedros contributed to the critical manuscript review and revision. Dimitris N. Tatakis contributed to the study design, critical review, and manuscript revision.

## Funding

This study did not receive any specific grant from funding agencies in the public, commercial, or not‐for‐profit sectors.

## Supporting information


**Supporting Information** Additional supporting information can be found online in the Supporting Information section. This case series was prepared in accordance with the CARE‐Checklist‐English (2013) reporting guidelines.

## Data Availability

The data supporting the findings of this study are available from the corresponding author, Dr. Najla S. Kasabreh, upon reasonable request. Due to patient confidentiality and the University of Jordan′s institutional data protection policies, raw clinical data are not publicly shared.
