# Supplementary material for: One‐Year Clinical Evaluation of the Horizontal Mattress Sling Suture for Aesthetic Crown Lengthening
Source: Case Rep Dent. 2025 Dec 22;2025:9986106. doi: 10.1155/crid/9986106 (PMC12723188; doi:10.1155/crid/9986106)
Supplement: Supplementary file 1 — Supporting Information Additional supporting information can be found online in the Supporting Information section. This case series was prepared in accordance with the CARE‐Checklist‐English (2013) reporting guidelines. [file CRID-2025-9986106-s001.pdf]

CARE
case report guidelines

CARE Checklist of information to include when writing a case report

(cc) BY-NC-ND

Topic	Item	Checklist item description	Reported on Line
Title	1	The diagnosis or intervention of primary focus followed by the words "case report"	1-3
Key Words	2	2 to 5 key words that identify diagnoses or interventions in this case report, including "case report"	41-42
Abstract (no references)	3a	Introduction: What is unique about this case and what does it add to the scientific literature?	22-26
	3b	Main symptoms and/or important clinical findings	26-28
	3c	The main diagnoses, therapeutic interventions, and outcomes	28-36
	3d	Conclusion—What is the main "take-away" lesson(s) from this case?	36-39
Introduction	4	One or two paragraphs summarizing why this case is unique (**may include references**)	56-62, 82-96
Patient Information	5a	De-identified patient specific information.	100,107,146,159
	5b	Primary concerns and symptoms of the patient.	108-109,146,159-161
	5c	Medical, family, and psycho-social history including relevant genetic information	99-101
	5d	Relevant past interventions with outcomes	NA
Clinical Findings	6	Describe significant physical examination (PE) and important clinical findings.	109-110,147-150,161-163
Timeline	7	Historical and current information from this episode of care organized as a timeline	100-104,112-113, 148-149, 163-164, 171-172
Diagnostic Assessment	8a	Diagnostic testing (such as PE, laboratory testing, imaging, surveys).	102-104
	8b	Diagnostic challenges (such as access to testing, financial, or cultural)	NA
	8c	Diagnosis (including other diagnoses considered)	110, 150, 162-163
	8d	Prognosis (such as staging in oncology) where applicable	NA
Therapeutic Intervention	9a	Types of therapeutic intervention (such as pharmacologic, surgical, preventive, self-care)	112-133,148-151, 165-169
	9b	Administration of therapeutic intervention (such as dosage, strength, duration)	134-139
	9c	Changes in therapeutic intervention (with rationale)	NA
Follow-up and Outcomes	10a	Clinician and patient-assessed outcomes (if available)	139-143,152-156,170-175
	10b	Important follow-up diagnostic and other test results	139-143,152-156,170-175
	10c	Intervention adherence and tolerability (How was this assessed?)	139-143,152-156,170-175
	10d	Adverse and unanticipated events	NA
Discussion	11a	A scientific discussion of the strengths AND limitations associated with this case report	179-186, 201-203, 207-210, 241-253
	11b	Discussion of the relevant medical literature **with references**.	188-240
	11c	The scientific rationale for any conclusions (including assessment of possible causes)	184, 186, 238-240,
	11d	The primary "take-away" lessons of this case report (without references) in a one paragraph conclusion	261-266
Patient Perspective	12	The patient should share their perspective in one to two paragraphs on the treatment(s) they received	186
Informed Consent	13	Did the patient give informed consent? Please provide if requested	Yes ☒ No ☐
